# A Novel Mouse Model Reveals that Polycystin-1 Deficiency in Ependyma and Choroid Plexus Results in Dysfunctional Cilia and Hydrocephalus

**DOI:** 10.1371/journal.pone.0007137

**Published:** 2009-09-23

**Authors:** Claas Wodarczyk, Isaline Rowe, Marco Chiaravalli, Monika Pema, Feng Qian, Alessandra Boletta

**Affiliations:** 1 Dulbecco Telethon Institute (DTI) at Dibit, San Raffaele Scientific Institute, Milan, Italy; 2 Johns Hopkins University School of Medicine, Baltimore, Maryland, United States of America; Institute for Research in Biomedicine, Spain

## Abstract

Polycystin-1 (PC-1), the product of the *PKD1* gene, mutated in the majority of cases of Autosomal Dominant Polycystic Kidney Disease (ADPKD), is a very large (∼520 kDa) plasma membrane receptor localized in several subcellular compartments including cell-cell/matrix junctions as well as cilia. While heterologous over-expression systems have allowed identification of several of the potential biological roles of this receptor, its precise function remains largely elusive. Studying PC-1 *in vivo* has been a challenging task due to its complexity and low expression levels. To overcome these limitations and facilitate the study of endogenous PC-1, we have inserted HA- or Myc-tag sequences into the *Pkd1* locus by homologous recombination. Here, we show that our approach was successful in generating a fully functional and easily detectable endogenous PC-1. Characterization of PC-1 distribution *in vivo* showed that it is expressed ubiquitously and is developmentally-regulated in most tissues. Furthermore, our novel tool allowed us to investigate the role of PC-1 in brain, where the protein is abundantly expressed. Subcellular localization of PC-1 revealed strong and specific staining in ciliated ependymal and choroid plexus cells. Consistent with this distribution, we observed hydrocephalus formation both in the ubiquitous knock-out embryos and in newborn mice with conditional inactivation of the *Pkd1* gene in the brain. Both choroid plexus and ependymal cilia were morphologically normal in these mice, suggesting a role for PC-1 in ciliary function or signalling in this compartment, rather than in ciliogenesis. We propose that the role of PC-1 in the brain cilia might be to prevent hydrocephalus, a previously unrecognized role for this receptor and one that might have important implications for other genetic or sporadic diseases.

## Introduction

Autosomal Dominant Polycystic Kidney Disease (ADPKD) is one of the most common monogenic disorders, affecting approximately 1/500–1/1000 of the population [1]. The hallmark of the disease is bilateral renal cyst formation, although ADPKD is a systemic disorder affecting several other organs. In 85% of all ADPKD cases, germ line mutations in the *PKD1* gene are responsible for the development of the disease, whereas in the remaining 15% of cases, the *PKD2* gene is mutated. *PKD1* and *2* are ubiquitously expressed and the generally accepted explanation for the formation of cysts in only some organs is the “two-hit” model. According to this model, a second somatic mutation affecting the normally inherited allele occurs in the renal and bile duct epithelia, resulting in the loss of function of either *PKD1* or *PKD2* and causing the expansion of clonal cysts. Haploinsufficiency is also believed to play a role in some of the systemic manifestation of the disease, such as some of the cardiovascular defects [1].

Complete loss-of-function murine models for either *Pkd1* or *Pkd2* have shown that the loss of either gene results in embryonic lethality associated with a number of different phenotypes, including renal cystogenesis, skeletal and cardiac abnormalities [2], [3], vascular lesions such as haemorrhage and oedema [4], [5], and placental defects [3], [6], consistent with the ubiquitous expression of the two genes. Heterozygous mice are healthy and develop only a few renal cysts during their adult life, possibly due to a low rate of second-hit in the mouse kidney. In fact, conditional inactivation of the *Pkd1* gene in the kidney results in massive cyst formation, with a very variable phenotype depending on the timing of inactivation [7], [8].

Finally, mouse models carrying a reduction in *Pkd1* expression [9], or transgenic overexpression of the *Pkd1* gene [10] all result in renal cystogenesis, suggesting that the expression levels and/or appropriate subcellular localization of PC-1 are critical for its normal function.

The *PKD1* gene encodes Polycystin-1 (PC-1, app. 520 kDa), a highly glycosylated plasma membrane receptor consisting of a large (app. 3000 aa) extracellular N-terminal portion with a novel combination of protein-protein interacting motifs [11], [12], 11 transmembrane domains, and a short (198 aa) intracellular C-terminus for regulating signal cascades [13]–[19]. Full-length PC-1 can be cis-autoproteolytically cleaved at its GPS site (G-protein coupled receptor proteolytic site), generating a C-terminal fragment of approximately 150 kDa (CTF) and an N-terminal fragment of approximately 400 kDa (NTF), which remains tethered to the CTF [20], [21]. This cleavage occurs ubiquitously and was shown to be essential for normal PC-1 function both *in vitro* and *in vivo* [20], [21]. The *PKD2* gene encodes Polycystin-2 (PC-2) protein, a TRP-like channel that interacts with the intracellular C-tail of PC-1 through a coiled-coil domain to form a functional complex [22], thus explaining the almost identical clinical phenotype of ADPKD1 and ADPKD2.

PC-1 localizes at cell-cell junctions in association with E-cadherin/β-catenin [23], at desmosomes [24], focal adhesions [25], and in the primary cilium [26]–[28]. In addition, two additional distinct cleavages have been reported to occur in the intracellular C-terminal domain of PC-1, resulting in the release of a membrane-free fragment, which is able to translocate to the nucleus [29], [30]. While the expression of PC-1 in all of these subcellular locations is not surprising for such a large, complex, and highly post-translationally regulated receptor, it should be noted that there is a general consensus that studying the endogenous protein is challenging due to a series of factors including the low specificity of antibodies, low protein expression levels, and the high complexity of the protein. Characterization of the overexpressed protein in transgenic mice has been proposed as a way to gain insight into the protein's normal processing, localization, and function. However, the generation of a cystic phenotype in the transgenic mice models reported to date [10] demonstrates that this approach perturbs the physiological dosage/function of PC-1 and thus cannot be employed to study its normal function.

To overcome these limitations and facilitate the study of PC-1 *in vivo*, we have used targeted homologous recombination to generate an easily detectable epitope-tagged conditional allele of PC-1. We report here that our approach was successful in generating a fully functional and tagged endogenous PC-1 that can easily be tracked *in vivo*. We show strikingly strong staining in newborn brains in the ependymal and choroid plexus (CP) ciliated cells. Defective ciliary function in these compartments has been linked to defective secretion (by the epithelia of the CP) or movement (by the beating of the ependymal cilia) of cerebrospinal fluid (CSF), ultimately causing dilatation of the brain ventricular system and hydrocephalus [31]–[38]. Consistent with this and the localization of PC-1 in brain tissue, we have found that inactivation of the *Pkd1* gene using either a ubiquitous Cre or a Cre expressed in the central nervous system results in hydrocephalus, a relatively common condition in humans.

Our data describe a previously unrecognized phenotype in the *Pkd1* mutant mice and extend the understanding of the ciliary function of PC-1 to a part of the body other than the kidney.

## Results

### Generation of epitope-tagged *Pkd1* knock-in mouse lines

**Figure 1:**
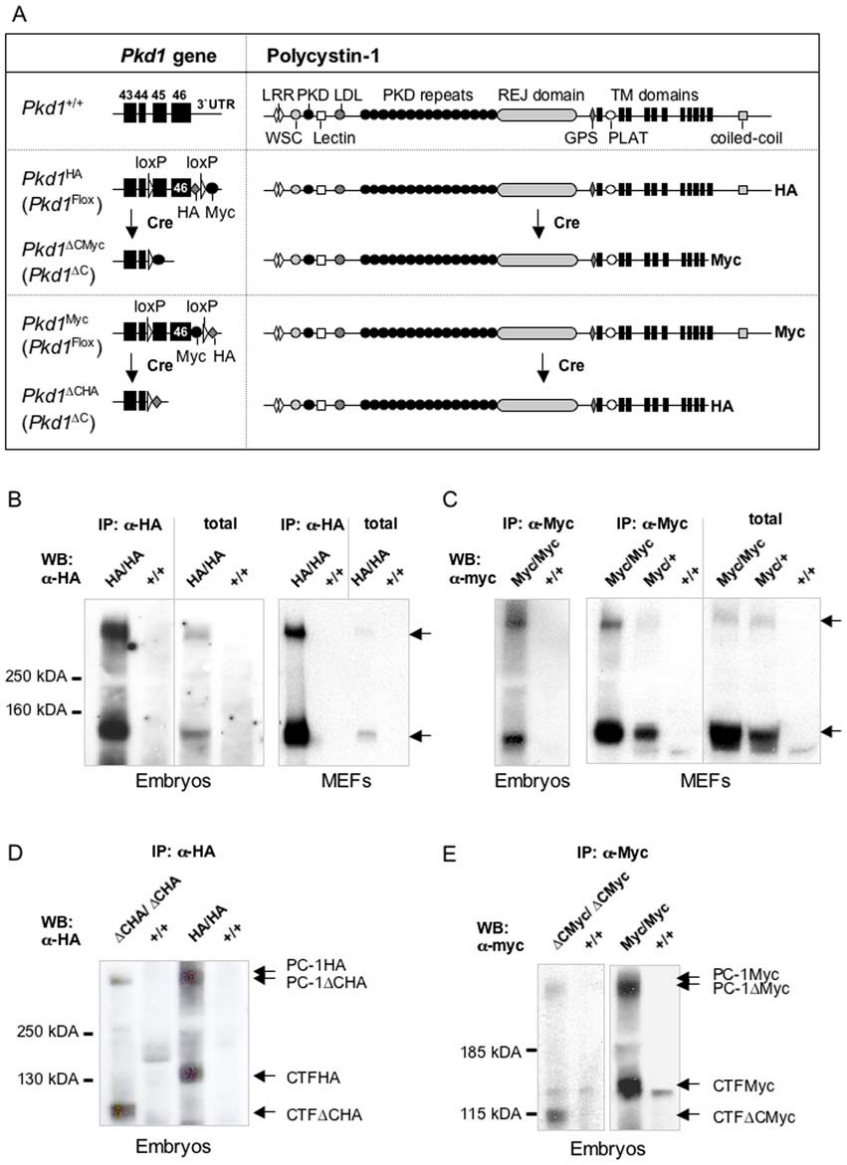
Generation of knock-in mouse lines and detection of tagged PC-1 in embryos and MEF cells. (A) Schematic overview of Polycystin-1 and the 3′region of the *Pkd1* gene in wild-type and knock-in mice before and after recombination with Cre. By Cre-mediated excision of the last two exons, a truncated PC-1 molecule carrying a tag is expected. (B, C) Homozygous *Pkd1^HA/HA^* (B) or *Pkd1^Myc/Myc^* (C) as well as wild-type littermate embryos (B: E14.5, C: E15.5) and MEF cells were analyzed by western blot. The expression of both full length (∼520 kDA) and cleaved (∼150 kDA) forms of tagged PC-1 were detected both by α-HA and by α-Myc antibodies. (D, E) Heterozygous knock-out lines were generated by crossing heterozygous knock-in lines with ubiquitous Cre-expressing mice [48]. After intercrossing, homozygous *Pkd1* mutants (D) *Pkd1* ^Δ*CHA/*Δ*CHA*^, (E) *Pkd1* ^Δ*CMyc/*Δ*CMyc*^, and wild type *Pkd1^+/+^* (negative control) embryos at E14.5/15.5 were isolated. Proteins were used for IP and analyzed by western blot. Immunoprecipitated tagged PC-1 of knock-in (D) *Pkd1^HA/HA^* and (E) *Pkd1^Myc/Myc^* embryos from corresponding developmental time points served as the positive control. The uncleaved (∼490 kDA) and cleaved (∼120 kDA) form of the truncated tagged PC-1 were detected (arrows). By comparing the full-length form (∼520 kDA) and the CTF (∼150 kDA) of the tagged PC-1 from knock-in embryos, a shift in size (−30 kDA) by excision of *Pkd1* exons 45/46 is visible.

In the attempt to generate a mouse model that would facilitate the study of endogenous Polycystin-1, we have generated two new mice lines carrying epitope-tagged Polycystin-1 by inserting either HA or Myc tags in frame to exon 46 of the *Pkd1* gene. In order to enhance the signal, several copies of both HA (3X) or Myc (5X) were inserted in the two targeting constructs (Figure 1A and Figure S1). In previous studies, we had found that similar modifications do not change the functional properties of PC-1 in a series of *in vitro* functional assays conducted on over-expressed protein [13], [19] and unpublished.

To avoid long-range sequencing reactions of the homologous arms of the targeting vectors, we used homologous recombination in bacteria (see Supporting Information Methods S1; 39) and generated a vector containing the sequences between *Pkd1* exon 39 and *Tsc2* exon 29 in which we stepwise inserted: I) recognition sites for Cre recombinase (loxP) in intron 44 and in the 3′UTR; II) HA (or Myc) epitopes in frame to exon 46 followed by a STOP codon; III) multiple Myc epitopes downstream the 3′ loxP site; IV) a neomycin cassette flanked by Frt sites in the 63 bp inter-region between the 3′ UTR of the *Pkd1* gene and the 3′UTR of the *Tsc2* gene (Figure S1). The relevant regions carrying the modifications (i.e. *Pkd1* intron 44, exon 46 and 3′UTR, and the *Pkd1/Tsc2* inter-region) were fully sequenced.

The two constructs, named HALM and MLHA (Figure S1) are expected to generate a HA-tagged and a Myc-tagged PC-1, respectively (Figure 1A). In addition, upon recombination with Cre, a truncated form of PC-1 lacking the C-terminus should be generated (Figure 1A). Furthermore, a second tag was inserted in the 3′UTR sequence so that upon Cre recombination it would become in frame to label the truncated PC-1 protein and generate a Myc-tag (*Pkd1* ^ΔCMyc^) or a HA-tag (*Pkd1* ^ΔCHA^), respectively, allowing us to track the mutant protein. Both constructs were used to generate ES cells carrying the desired modifications and the positively selected clones were used to generate chimeras and subsequently the *Pkd1* ^HAneo/+^ and *Pkd1* ^Mycneo/+^ lines from which the neo cassette was removed to generate *Pkd1* ^HA/+^ and *Pkd1* ^Myc/+^ lines (see Supporting Information).

We first examined whether we were able to detect tagged endogenous PC-1. To this end, heterozygous *Pkd1* ^HA/+^ or *Pkd1* ^Myc/+^ mice were intercrossed and homozygous *Pkd1* ^HA/HA^ or *Pkd1* ^Myc/Myc^ embryos were generated. Total lysates from the embryos or from murine embryonic fibroblasts (MEFs) isolated at day E11.5 were generated and analyzed for PC-1 expression either by western blot or by immunoprecipitation. In Figures 1B and C we show that endogenous full-length PC-1 (∼520 kDa) as well as the CTF cleavage product (∼150 kDa; 20) could be detected both in total lysates and after immunoprecipitations with anti-HA in the *Pkd1* ^HA/HA^ and with anti-Myc antibodies in the *Pkd1* ^Myc/+^ or *Pkd1* ^Myc/Myc^ cells and tissues (Figures 1B and C).

### The epitope-tagged *Pkd1* knock-in mouse lines contain fully functional PC-1 protein

To exclude the possibility that the inserted modification might generate a hypomorphic allele, we tested if the tagged form of PC-1 was able to rescue embryonic lethality in *Pkd1* knock-out mice. To this end, we crossed our knock-in/conditional knock-out mice lines with a mouse line carrying ubiquitous Cre recombinase (Figure 1A). We observed that inactivation of the *Pkd1* gene generated a truncated tagged protein lacking the C-terminal domain (Figures 1D and E) and resulting in a phenotype very similar to the one described for all other knock-out lines, including oedema, haemorrhage, and cystic kidneys (Figure S2) by day E15.5 [2]–[6], [40]. However, *Pkd1* ^ΔC/ΔC^ appear to have a milder phenotype as compared to the complete null mouse model, since in *Pkd1* ^ΔC/ΔC^ lines analyzed more than 90% of *Pkd1* ^ΔC/ΔC^ mice survived to E15.5 and approximately 60% survived to day E16.5 (Table S1) on a pure C57BL/6 background, whereas complete null [40] mice die at day E12.5 in a C57BL/6 background (Chiaravalli, Wodarczyk, Germino and Boletta, unpublished).

Next, we performed rescue experiments by crossing heterozygous *Pkd1* ^HA/+^ and *Pkd1* ^Myc/+^ mice with the heterozygous *Pkd1* knock-out line *Pkd1* ^+/ΔC^. *Pkd1* ^HA/ΔC^ and *Pkd1* ^Myc/ΔC^ pups were born at the expected mendelian ratio (Table 1), were viable and fertile and did not show any appreciable difference in renal, hepatic, or pancreatic cyst formation at 18 months of age as compared to *Pkd1* ^+/−^ mice (not shown). In addition, the *Pkd1* ^HA/HA^ and the *Pkd1* ^Myc/Myc^ double homozygote mice did not show any renal or extra-renal defects even with aging (>18months). These data strongly suggest that the modifications inserted did not generate a grossly hypomorphic allele.

**Table 1:** Overview of intercrosses between heterozygous knock-in/knock-out lines.

	***Pkd1*** **^HA/ΔC^**	***Pkd1*** **^HA/+^**	***Pkd1*** **^+/ΔC^**	***Pkd1*** **^+/+^**	**total**
Nr.:	12 (20%)	15 (25%)	19 (31.7%)	14 (23.3%)	60
	***Pkd1*** **^Myc/ΔC^**	***Pkd1*** **^Myc/+^**	***Pkd1*** **^+/ΔC^**	***Pkd1*** **^+/+^**	**total**
Nr.:	24(1^†^)(30.4%)	13 (16.5%)	23 (29.1%)	19 (24%)	79(80)

To clarify whether the modifications in the *Pkd1* knock-in lines generated a hypomorphic allele, the heterozygous knock-in lines *Pkd1^HA/+^* and *Pkd1^Myc/+^* were bred with *Pkd1* ^*+/*Δ*C*^. Bi-transgenic animals were born and were healthy.

### Biochemical analysis of PC-1

Next, in order to control that the tags added onto the PC-1 C-terminus do not affect its ability to interact with other proteins, we investigated the interaction between PC-1 and PC-2, which is known to be mediated by the PC-1 C-terminus [22].

**Figure 2:**
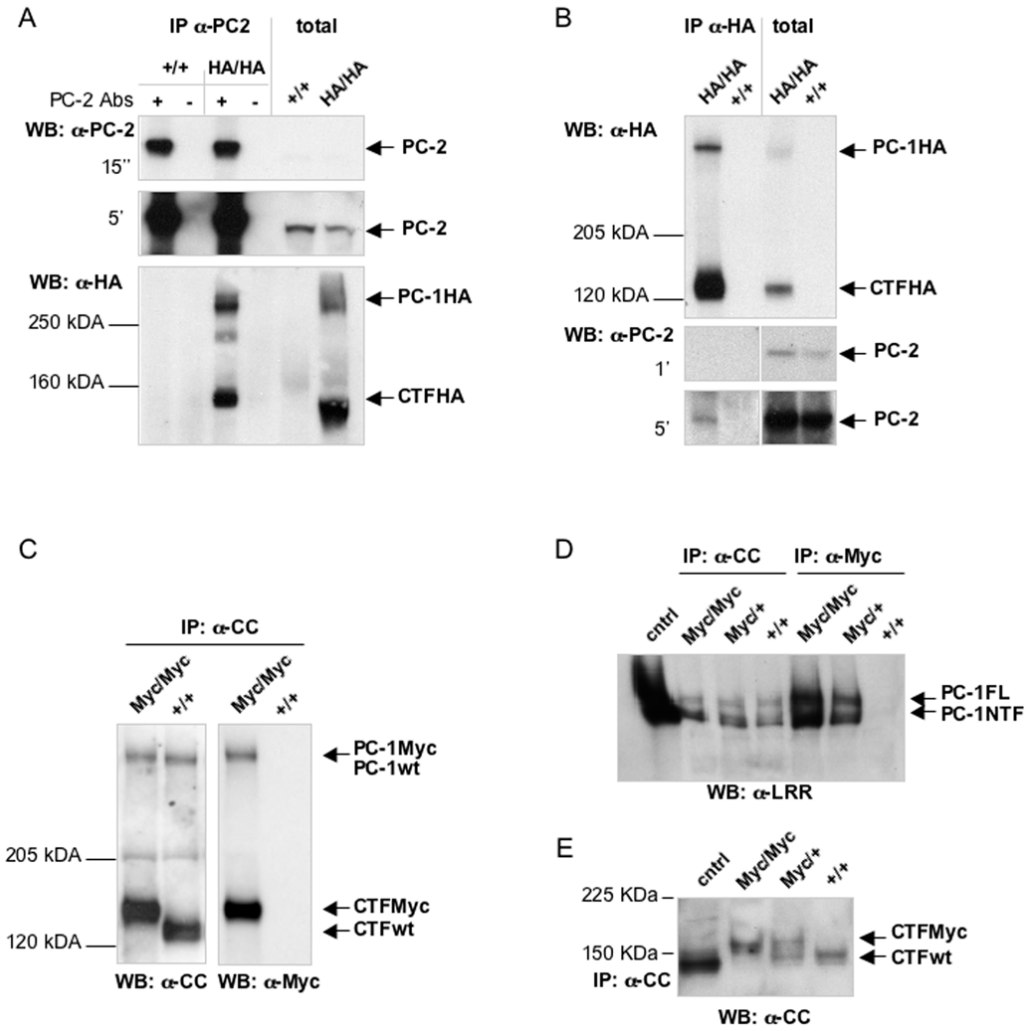
Biochemical characterisation of tagged endogenous PC-1. (A, B) To exclude that the tags at the C-terminus of PC-1 affect the interaction with other proteins, the interaction between HA tagged PC-1 and endogenous PC-2 was investigated. Proteins from homozygous knock-in *Pkd1^HA/HA^* and wild type MEF cells were isolated. (A) IP with α-PC-2 antibodies (PC-2 +) and unrelated IgGs as a control (−) was performed. PC-2 co-immunoprecipitated HA-tagged PC-1, showing that the PC-1/PC-2 interaction occurs normally in knock-in mice. (B) Immunoprecipitation using α-HA antibodies followed by western blot with anti-HA or anti-PC-2 antibodies confirmed the reciprocal interaction of tagged endogenous PC-1 with endogenous PC-2. (C, D, E) To compare the expression levels of tagged versus wild-type endogenous PC-1, proteins from knock-in and wild-type MEFs (C) and E14.5 embryos (D, E) were isolated. (C) PC-1 was immunoprecipitated by antibodies directed against the C-terminus of PC-1 (anti-CC), and analyzed by western blot using either anti-CC (left panel, see material and methods) [21] or anti Myc antibodies (right panel). We observed equal expression levels of tagged and endogenous PC-1 (full length as well as CTF) in MEFs isolated from Pkd1^Myc/Myc^ and Pkd1^+/+^ embryos using anti-CC antibodies (left panel). (D) Samples immunoprecipitated with α-CC or α-Myc antibodies followed by western blot analysis using a α-LRR antibody confirmed that equal expression levels of the full-length tagged and wild-type endogenous protein could be detected by α-LRR (PC-1FL). Furthermore, the amount of NTF tethered to the CTF (PC-1NTF) was identical in the two species. As expected, immunoprecipitation with α-Myc was more efficient than with α-CC (right lanes). Ctrl: positive control represented by over-expression of recombinant PC-1. (E) Comparing the tagged and endogenous PC-1 in heterozygous *Pkd1^Myc/+^* embryos revealed that the two alleles result in identical expression levels of the CTF in tagged versus wild-type endogenous PC-1. Ctrl: positive control represented by over-expression of recombinant, untagged PC-1.

First, protein lysates from *Pkd1* ^HA/HA^ and control wild type MEFs were immunoprecipitated using an anti-PC-2 antibody followed by immunoblotting with anti-HA. In Figure 2A, we show that PC-2 is able to immunoprecipitate endogenous, HA-tagged PC-1 both in its full-length and CTF forms. Next, we performed the reverse experiment and used anti-HA antibodies to immunoprecipitate endogenous, tagged PC-1 from mouse embryonic fibroblasts. Western blot analysis show that PC-2 is immunoprecipitated along with PC-1 in a specific manner, since an IP using anti-HA antibodies in lysates from wild-type MEFs did not show any PC-2 immunoprecipitation, despite the fact that equal amounts of PC-2 are expressed by both cell lines (Figure 2B).

Next, in order to compare the expression levels and biochemical processing of the tagged protein, we used recently developed antibodies directed against murine PC-1 [21] to immunoprecipitate PC-1 from wild-type and *Pkd1* ^Myc/Myc^ fibroblasts and embryos to compare the expression levels. In Figures 2C and D we show that equal levels of full-length PC-1 were detected in *Pkd1* ^+/+^ and *Pkd1* ^Myc/Myc^ fibroblasts and embryos. Likewise, the extent of cleavage at the GPS site and the amount of NTF cleavage product remaining tethered to the CTF fragment were indistinguishable between the wild-type and the tagged proteins (Figure 2D). Interestingly, the CTF cleavage product observed in the *Pkd1* ^Myc^ lines migrated at a slightly slower rate in SDS-page due to the larger size of the protein generated by the tags distinguishing the tagged from the untagged form of the protein in a single lane. We therefore took advantage of this feature to perform a direct comparison of the expression levels of the tagged versus untagged protein using *Pkd1* ^Myc/+^ embryos. Figure 2E shows that immunoprecipitation with an anti-PC-1 C-terminal antibody followed by immunoblotting reveals that there are equal amounts of tagged versus untagged CTF in these lysates. All of these data taken together strongly suggest that the tagged protein behaves like the endogenous untagged protein.

### Characterization of PC-1 Expression and Distribution in the Kidney

**Figure 3:**
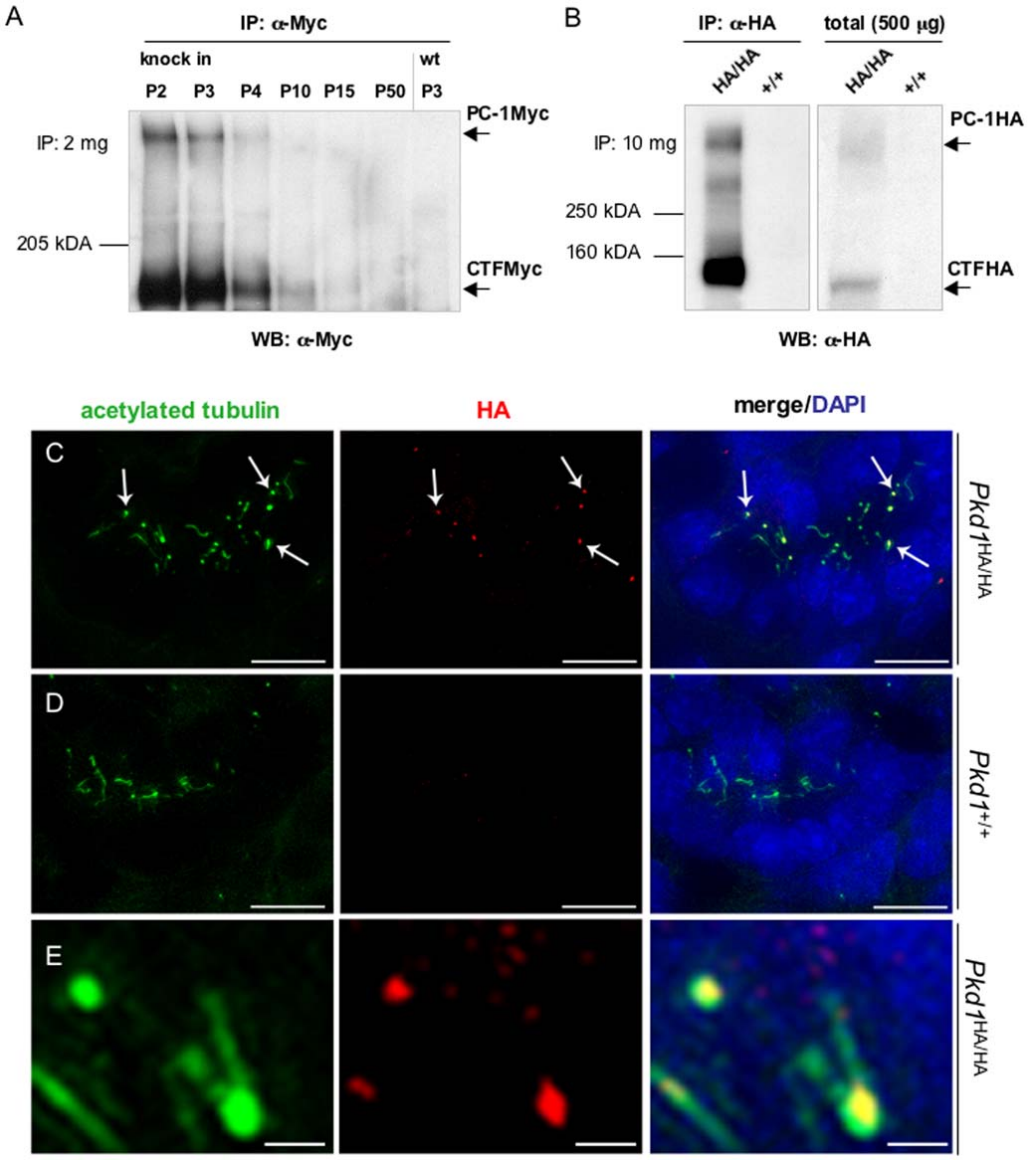
Expression of tagged endogenous PC-1 in the kidney. (A) Kidneys from homozygous *Pkd1^Myc/Myc^* mice were isolated, and Myc tagged PC-1 at different time points between P2 and P20 was immunoprecipitated by α-Myc specific antibodies. Western blot analysis shows a dramatic decrease in the expression level of PC-1 between P4 and P10. (B) Kidneys from *Pkd1^HA/HA^* mice at P20 were isolated and expression of PC-1 was assessed by immunoprecipitation from 10 mg of total protein lysates followed by western blot analysis. The protein was also visible in total lysates if 500 µg of total lysates was loaded in a single lane (right blot). (C, D, E) Kidneys from homozygous *Pkd1^HA/HA^* (C) as well as wild-type (D) newborns at P2 were isolated, dissected, and immunostained with α-HA antibodies for detection of HA-tagged PC-1 (red), and acetylated α-tubulin for cilia staining (green). The staining in C shows localization of PC-1 at the basis of cilia in the kidneys of *Pkd1^HA/HA^* mice. This staining is completely absent in Pkd1^+/+^ kidneys, demonstrating its specificity. (E) Higher magnification of the immunostaining in C shows that PC-1 localizes to the base of cilia. Scale bars: C,D 10 µm; E 2 µm.

We first investigated the expression of PC-1 in the kidney. Studies in mice have previously shown that PC-1 expression decreases during the postnatal period [21], [41]. We therefore assessed the expression of PC-1 at different time points starting in newborn kidneys and up to P50. As shown in Figure 3A, PC-1 appears to be highly expressed in the newborn kidney at day P2 and it appears that there is a very sharp time window between days P4 and P10 in which PC-1 expression levels drop drastically. Immunoprecipitation using a much higher amount of total protein lysate (10 mg) showed the presence of PC-1 in the adult kidney (P20) (Figure 3B), although the protein appears to be expressed at much lower levels.

We therefore performed immunofluorescence studies in newborn kidneys (P2) from homozygous *Pkd1* ^HA/HA^ mice using an anti-HA antibody. We detected the presence of bright single spots on the apical side of epithelial cells at the base of cilia, as evidenced by counter-staining with anti-acetylated alpha-tubulin. A similar bright staining pattern was also present in the absence of acetylated tubulin counterstaining. These results are consistent with previously reported studies [27], [28] further confirming that the tagged form of PC-1 behaves like the endogenous protein (Figures 3C–E). Staining of control, wild-type sections in identical conditions showed that the staining is specific since anti-HA antibodies were not able to detect cilia in the wild-type mice (not shown). In addition, very similar staining was observed by staining *Pkd1* ^Myc/Myc^ embryos using an anti-Myc antibody.

### PC-1 localizes to ependymal and choroid plexus cilia

**Figure 4:**
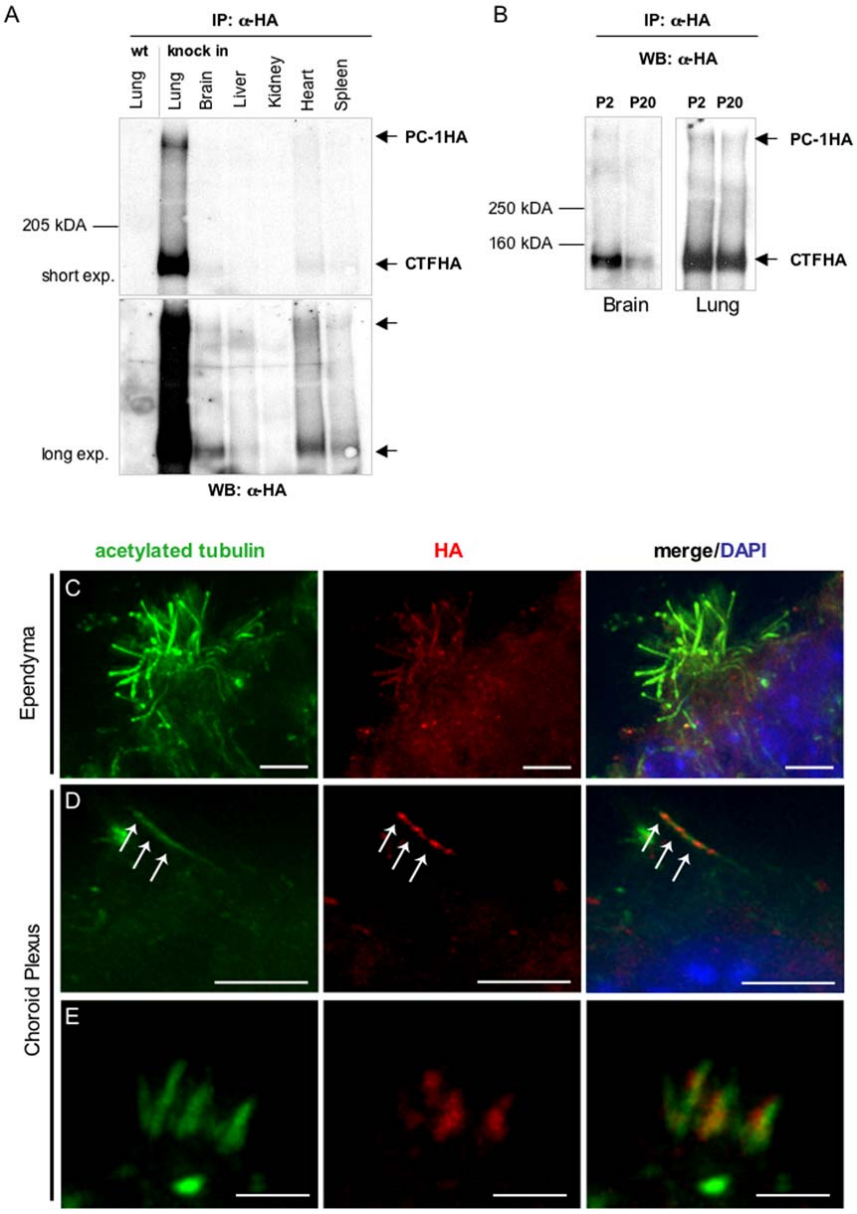
Expression of tagged PC-1 in brain and adult tissues. (A, B) Different tissues from homozygous *Pkd1^HA/HA^* mice (P2 and P20) were isolated and analyzed by IP and western blots. (A) Different exposure times of the same western blot revealed that the expression of PC-1 is very low in kidney, moderate in the brain, heart, liver and spleen, and very high in the lung. (B) PC-1 expression levels were compared in lung and brain at P2 and P20 by IP/western blot. Whereas PC-1 is constantly expressed at high levels in the lung, in the brain the expression levels decrease between days 2 and 20 of postnatal life of the mouse. (C–E) Brains of homozygous as well as wild-type litter mates from P2 were isolated, dissected, and immunostained with anti-HA antibodies for detection of HA-tagged PC1 (red) and acetylated α-tubulin for labelling the cilia (green). Figure 4C shows the staining of ependymal multiciliated cells with anti-HA antibodies. (D, E) Both monociliated (D) and multiciliated (E) choroid plexus cilia stained positive with anti-HA antibodies. Scale bars: C,D 5 µm; E 2 µm.

We next examined the expression of PC-1 in several organs at P20. Tissues from homozygous *Pkd1* ^HA/HA^ as well as control wild-type littermates were isolated and used in immunoprecipitation experiments using anti-HA antibodies. As shown in Figure 4A, expression in the lung is much higher than in any other organ. PC-1 expression was also detected in the brain, heart, and spleen. As noted above, expression of PC-1 in P20 kidneys appeared to be much lower than in any other organ, although detectable (Figure 3B)

We thus analysed if expression in the brain and lung was subject to postnatal regulation similar to what was observed for the kidney. We found that PC-1 expression in brain appears to be diminished at day P20 compared to P2, while expression in the lung appears to be constant throughout the life of the mouse (Figure 4B).

We next focused on PC-1 distribution in brain, since this receptor was never characterized in this compartment. To this end, we analyzed the distribution of PC-1 in brain by immunofluorescence studies on early postnatal tissue (P2), where expression appears to be reasonably strong (Figure 4B and data not shown). Analysis of brain sections from *Pkd1* ^HA/HA^ and *Pkd1* ^Myc/Myc^ mice as well as littermate wild-type control mice revealed the presence of PC-1 in ciliated structures. Counterstaining with anti-acetylated alpha tubulin confirmed immunolocalization of PC-1 in all types of cilia observed in the lateral and third ventricles [31], including mono- as well as multiciliated choroid plexus cells and multiciliated ependymal cells. Identical results were seen by analyzing the distribution of the HA- or Myc-tagged protein (Figures 4C–E and Figure S3). Neither anti-HA nor anti-Myc antibodies detected any positive signal in wild-type tissues, demonstrating the specificity of the antibody (Figure S3). Furthermore, staining with anti-HA or anti-Myc antibodies in the absence of anti-acetylated alpha-tubulin counterstaining revealed an identical staining pattern, demonstrating that the signal is not generated in a non-specific manner by the intense staining of cilia (not shown).

At higher magnification, we discovered profound differences in the distribution of PC-1 depending on the type of cilia analyzed. In grouped cilia present in the ependyma, PC-1 was concentrated either at the tip or along the axoneme of the cilia (Figure 4C). In primary cilia of the choroid plexus (Figure 4D), PC-1 was clearly present along the ciliary axoneme with spotty staining both in the HA and in the Myc lines, resembling the distribution observed for IFT particles. We also observed bundles of cilia in the choroid plexus exhibiting a shorter and more compact structure, as described previously [32]. In these types of cilia, PC-1 appeared to be present at the base of cilia (Figure 4E). These results suggest that PC-1 might exert different functions on different cilia. Alternatively, since the tags are inserted into the C-terminus of PC-1 and we are therefore able to detect all of the cleavage products containing the C-tail, it is also possible that the different cleavage products are distributed differently in the cilia.

### *Pkd-1* Knock-Out Mice Show Signs of Mild Hydrocephalus by E16.5

**Figure 5:**
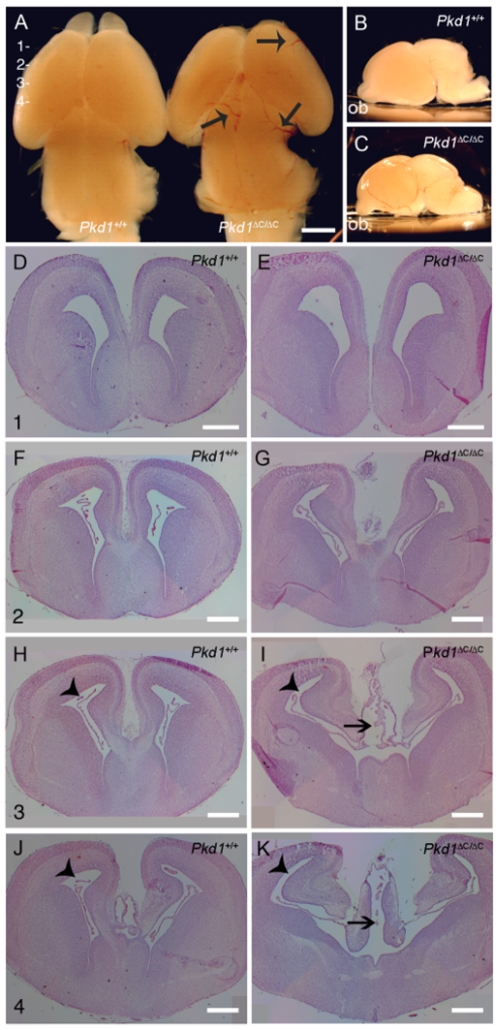
*Pkd1* knock-out embryos show mild hydrocephalus at E16.5. Upper view (A) and lateral view (B, C) of wild-type and knockout brains at E16.5 show macroscopic differences with dilated vessels (arrows) and olfactory bulbs (ob) pointing down. Coronal sections corresponding to 1 (D, E), 2 (F, G), 3 (H, I), and 4 (J, K) show that the third ventricle (arrow) from the mutant brains is strongly dilated compared to the wild-type. The lateral ventricle was only mildly dilated. (arrowhead). All the images are representative of an identical phenotype observed in 8 different mutant mice and never observed in 8 different wild-type controls. Scale bar A 1 mm; D to G 500 µm.

Since we found strong ciliary staining both in ependymal and choroid plexus cilia, we wondered whether the absence of Polycystin-1 in these structures would cause an overt phenotype or if PC-1 function in this compartment is redundant and compensated for by other factors, given that a defect at this level was never reported before in *Pkd1* mutant mice. To answer this question we analyzed the brains of *Pkd1* ^ΔC/ΔC^ mice compared to the wild-type littermates at day E16.5. As shown in Figure 5, the brains of wild-type and *Pkd1* ^ΔC/ΔC^ mice appear macroscopically different, with the two separated frontal lobes (Figure 5A) and several dilated vessels (Figure 5A, arrows; n = 8). Lateral views show that the brains of *Pkd1* ^ΔC/ΔC^ mice appeared more curved, with the olfactory bulbs (ob) pointing down due to the dome-shape of the brain (Figures 5B and C, ob; n = 8). Morphological analysis of sections showed a mild dilatation of the lateral ventricles and a more prominent dilatation of the third ventricle, with a considerable defect in choroid plexus (CP) morphology (Figure 5D–K, n = 5). Interestingly, the aqueduct of Sylvius does not show obvious signs of stenosis, excluding the possibility of ventricle dilatation as a secondary cause of aqueduct stenosis (not shown). The images shown in figure 5A–C are representative of an identical phenotype observed in eight distinct mutant mice (100% penetrance) and completely absent from eight distinct littermate wild type controls withdrawn at the same time under identical conditions, thus excluding the possibility that the defects observed might be secondary to problems in dissection. Likewise, the images in figure 5D–K are representative of five distinct mutant and wild-type littermate controls subject to cross-sections showing an identical phenotype.

### Conditional inactivation of *Pkd1* in brain results in postnatal hydrocephalus

**Figure 6:**
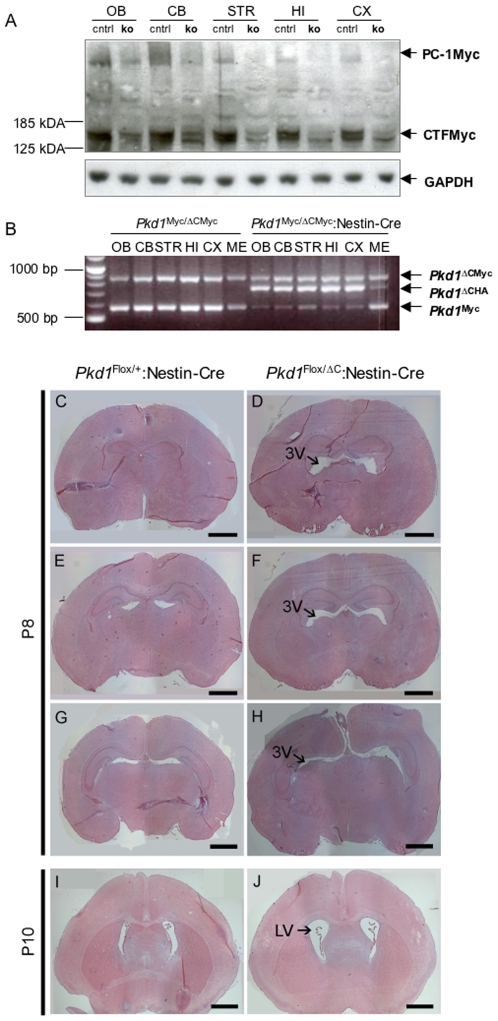
Conditional inactivation of *Pkd1* in the brain results in hydrocephalus. Knock-in *Pkd1^Myc/Myc^* mice were bred with a Nestin-Cre line to generate *Pkd1^Myc/+^;Nestin-Cre* mice. This line was next crossed with heterozygous mutant *Pkd1* ^*+/*^^Δ*CMyc*^ mice, to generate brain conditional mutant mice *Pkd1^Myc/^* ^Δ*Myc*^ *;Nestin-Cre* and *Pkd1^Myc/^* ^Δ*Myc*^ (control) littermates. (A, B) Brains from P7 mice were isolated and different regions were separated (OB: Olfactory bulb, CB: Cerebellum, STR: Striatum, Putamen, HI: Hippocampus, CX: Cortex, ME: Meninges). (A) By western blot analysis using anti-Myc antibodies, a reduction in the expression of full-length Myc-tagged PC-1 in conditional knock-out mice *Pkd1^Myc/^* ^Δ*CMyc*^ *;Nestin-Cre* could be seen compared to *Pkd1^Myc/^* ^Δ*Myc*^ control lines not expressing the Nestin-Cre transgene. GAPDH staining was used as a loading control. (B) The excision of *Pkd1* exons 45/46 by Cre recombinase was analyzed by PCR. In samples from the brain of control *Pkd1^Myc/^* ^Δ*CMyc*^ mice, the knock-in *Pkd1^Myc^* (519 bp) and knock-out *Pkd1* ^Δ*CMyc*^ (862 bp) alleles were detected. In conditional knock-out brain of *Pkd1^Myc/^* ^Δ*CMyc*^ *;Nestin-Cre* mice, the appearance of an additional PCR product of 748 bp was observed identifying the excised allele. The excision occurred in all investigated brain regions and, as expected, to a much lesser extent in the meninges. (C–H) Coronal sections of *Pkd1^Flox/^* ^Δ*C*^ *;Nestin-Cre* brains (D, F, H) and the control *Pkd1^Flox/+^;Nestin-Cre* brains (C, E and G) at P8 stained with Hematoxylin-Eosin show hydrocephalus with dilatation of the third ventricle (arrow). (I, J) Coronal sections of *Pkd1^Flox/^* ^Δ*C*^ *;Nestin-Cre* brains (J) and the control *Pkd1^Flox/+^;Nestin-Cre* brains (I) at P10 show dilatation of the lateral ventricles (arrow). Scale bar C–J 1 mm.

To follow the development of hydrocephalus at later time points we crossed our *Pkd1* ^Flox^ mice with a line carrying Cre recombinase in a restricted expression pattern to overcome the embryonic lethality of the *Pkd1* ^ΔC/ΔC^ mice, but with a broad expression pattern in the brain to allow targeting of all the regions of the CNS. We used the Nestin-Cre mouse line previously employed to inactivate Stumpy, another ciliary protein reported to cause hydrocephalus when inactivated [33], [42]. First of all, we analyzed the expression levels of Polycystin-1 in the different brain regions at P7 and found that the protein is expressed equally and abundantly in all regions (Figure 6A and not shown). Next, we compared the expression levels of the protein in *Pkd1* ^Myc/ΔCMyc^ in the presence or absence of Cre recombinase, and found that inactivation of the *Pkd1* gene and reduction of the PC-1 protein occurring at equal rates in all of the brain regions examined (Figures 6A and B). Most importantly, analysis of brain sections derived from Nestin-Cre conditional mouse models revealed the presence of hydrocephalus with dilation of the third ventricle at day P8 (Figure 6C–H, n = 2), which was subsequently followed by the dilation of the lateral ventricles, showing a mild but typical triventricular phenotype by day P10 (Figure 6I–J, n = 3). As previously reported, the Nestin-Cre transgene is also active in the kidneys [33]. In agreement with this, the *Pkd1* ^Flox/ΔC^;Nestin-Cre mouse developed renal cystogenesis and died of kidney failure around P12 (not shown). This prevented us from determining if a more severe phenotype would develop at later time points. In all of these experiments the Nestin-Cre transgene was employed as a heterozygous allele, since homozygosity of a similar transgene was previously reported to cause hydrocephalus itself [43]. In addition, *Pkd1* ^Flox/+^;Nestin-Cre brains were used as negative controls (Figures 6C, E, G and I) and none developed any sign of hydrocephalus, excluding the possibility that the transgene alone induced this phenotype in the heterozygous state. The ventricular dilations observed are very similar to the phenotype present in the null embryo and were again found with 100% penetrance (n = 5).

### Cilial morphology and apico-basal polarity in the ependyma and choroid plexus

**Figure 7:**
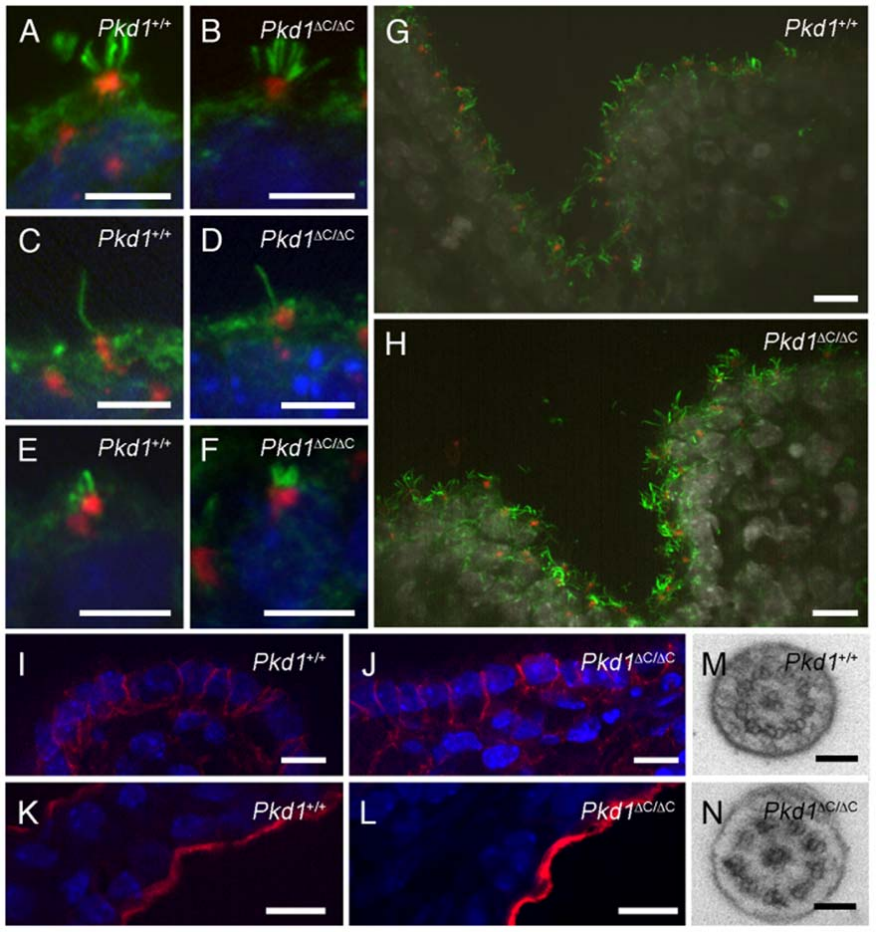
Cilia morphology and apico-basal polarity in the brain from knock-out embryos at E16.5. (A to H) Centrosomes and cilia in the choroid plexus from E16.5 brain were stained with anti-pericentrin (red) and anti-acetylated alpha-tubulin antibodies (green), respectively. Multicilia, monocilia, and bundles from lateral ventricles in knock-out (B, D, and F, respectively) and in wild-type (A, C, E) do not show differences in shape or number. Cilia from choroid plexus of the third ventricle of wild-type (G) and knock-out (H) animals do not show any differences. Staining of β-catenin of the choroid plexus in the third ventricle (I and J) and Aquaporin-1 in the choroid plexus of the lateral ventricle (K, L) did not reveal major differences. Transverse sections of the cilium of an ependymal cell from the third ventricle of wild-type (M) or mutant (N) animals carried out by electronic microscopy revealed the normal structure of the cilium. Scale bar A to F 5 µm; G to L 10 µm; M and N 100 nm.

Hydrocephalus is a condition associated with dilatation of the brain ventricles caused by accumulation of the cerebrospinal fluid which is either: I) secreted in excess by the choroid plexus; II) not properly propelled through the aqueduct and ventricles due to a failure of the ependymal cilia beating or stenosis of the aqueduct; III) improper re-absorption by the archnoid villi [31], [34], [37]. In previous mouse models of hydrocephalus due to mutations of ciliary proteins a dual mechanism of hydrocephalus formation due to both lack of proper cilia beating and defective fluid secretion, possibly regulated by cilia themselves, was proposed [34], [37]. We therefore analysed the morphology of cilia in E16.5 brains and found no differences in the shape of different cilia types on the choroid plexus (Figures 7A–H), nor in their relative numbers (not shown). In addition, electron microscopy analysis showed the normal 9+2 structure of the cilia of ependymal cells of the third ventricle (Figures 7M–N). These data are in agreement with the previously proposed role of Polycystin-1 in ciliary function rather than in ciliogenesis [27], [28]. Since defects in the apicobasal polarity of epithelial cells has been reported in the renal cystic epithelium of ADPKD patients, we wondered whether major defects in the morphology of the choroid plexus epithelium might account for the phenotype observed. Staining with Aquaporin-1, a marker of apical membrane, or beta-catenin, a marker of cell-cell junctions, revealed that no major differences could be observed in the choroid plexus epithelia between *Pkd1* wild-type and knock-out brains (Figures 7I–L).

## Discussion

In this report, we present the generation and characterization of two novel knock-in mouse models carrying either HA or Myc-tagged endogenous Polycystin-1. We have shown that the manipulations required to generate these alleles do not alter the function of PC-1, since both tagged alleles were able to rescue the embryonic lethality caused by inactivation of the *Pkd1* gene, and adult heterozygous mice carrying the tagged-allele (*Pkd1* ^HA/−^ or *Pkd1* ^Myc/−^) have an identical incidence of renal, pancreatic, and liver cystic lesions to that observed in heterozygous animals carrying the wild-type allele (*Pkd1* ^+/−^). In addition, we have performed a series of biochemical studies to compare the tagged versus wild-type alleles and shown that both HA and Myc-PC-1 have an identical pattern of expression, cleavage, association with the NTF, and heterodimerisation with PC-2 as compared to wild-type protein. All of this evidence leads us to conclude that the endogenous tagged Polycystin-1 behaves identically to endogenous wild-type protein. In addition, we have shown that inclusion of either tag allows for much more efficient detection of PC-1, as we were able to visualize the endogenous protein in total lysates in the absence of an immunoprecipitation enrichment step, as well as by immunofluorescence. Furthermore, using this system, we were able to provide conclusive evidence of the interaction between endogenous PC-1 and PC-2. Thus, we expect our model system to enormously facilitate future studies on the biology of PC-1.

Notably, we have found that PC-1 is developmentally regulated with a dramatic downregulation of its expression in most tissues during the perinatal life, as was reported previously. In particular, expression in the kidneys is dramatically downregulated during a narrow window of time, between days P4 and P10. In addition, we have shown that expression of PC-1 is ubiquitous but found at very low levels in most adult tissues, including the kidney, liver, pancreas, muscle (not shown), heart, and brain. Dissection in the different areas of the brain revealed that PC-1 is expressed in all areas with slightly higher expression in the olfactory bulbs and cerebellum. Surprisingly, the organ showing the highest expression levels in the adult is the lung and we have attributed this to the absence of postnatal downregulation of PC-1 in the lung. Of great interest, a recent study has reported a bronchiectasis defect in ADPKD patients [44], suggesting that PC-1 function in lungs might be essential in humans.

In this study, we have focused our attention on characterization of PC-1 role in brain, where we were able to track the protein thanks to the novel system developed. This is not a compartment known to be affected in ADPKD, despite the reasonably high expression levels of PC-1. This is not surprising, but rather provides further support to the two-hit hypothesis, which predicts that renal cystogenesis generates as a consequence of loss of the normal copy of the *PKD1* gene. Thus, our data support the notion that loss of function of both copies of the *PKD1* gene are necessary in order for its function to be inactivated. Thus, based on our evidences it appears that either the *PKD1* gene does not undergo second-hits in the ependyma and choroid plexus, or else that its inactivation in a single cell in this compartment does not provide a growth advantage necessary for the phenotype to manifest.

However, our data also highlight the broad role of the PC-1 receptor in mammalian development, as previously suggested based on the severe phenotype observed in *Pkd1* mutant mice [2]–[5]. Therefore, although the brain is not an organ affected in ADPKD, we believe that studying PC-1 function in this compartment will help shedding light on fundamental features of this receptor, including its subcellular distribution and function. In this study we show that PC-1 can be detected in ependyma and Choroid Plexus (CP), where we found a strong and specific staining in all three types of cilia observed in this region of the brain, i.e., multiciliated and monociliated CP cells and multiciliated ependymal cells. We believe that the distribution observed in these cilia is very informative, as PC-1 appears to localize in a slightly different manner in the different type of cilia. In fact, PC-1 is present at the base of the cilium in the kidney and in tight bundles of multiciliated choroid plexus cells, whereas it distributes along the axoneme with an IFT-like appearance in the monociliated CP cells and it shows more diffuse axonemal staining in the multiciliated ependymal cells. These various distributions might represent differences in the functions of PC-1 or else in the speed of intraflagellar transport in the different cilia, with slower movement in the monociliated CP cells and a longer residence time at the base in the renal monocilia and in the tight multiciliated bundles of the CP. Whatever the explanation for these different staining patterns, our data are in agreement with a previous report showing that inactivation of Polaris, an IFT protein, in the Tg737 mouse results in accumulation of PC-1 at the tip of monocilia, consistent with its possible transportation along the axoneme [34]. Furthermore, studies in the biflagellate unicellular alga *Chlamydomonas reinhardtii* have shown that the *Pkd2* orthologue fused to GFP is able to move along the axoneme, further suggesting that the Polycystins can indeed be transported via intraflagellar transport along the ciliary axoneme [45].

Finally, we not only showed that PC-1 is localized to ependymal and choroid plexus cilia, we also showed a novel phenotype of *Pkd1* mice consistent with a function for PC-1 in these structures. In fact, we have observed a mild hydrocephalus both in *Pkd1* mutant embryos and in newborn conditional mice carrying a Nestin-Cre transgene. Our data suggest that the brain ventricular system is impaired in the absence of a functional *Pkd1* gene, most likely due to a developmental defect. During the last phases of embryonic development starting at E14.5 in the mouse, the ventricles, appearing as large cavities, start narrowing to become very compact in the newborn mouse [31]. This process appears to be impaired in the *Pkd1* mutant brain at E16.5, with a major defect in the third ventricle and a much milder effect in the lateral ventricles. These data are particularly interesting when placed in the context of ependymal cilia development. In fact, it was prteviously reported that ependymal cilia are not visible in the lateral ventricles of E16.5 brains, while they are already detectable in the third ventricle [46]. At later time points during development, ependymal cilia become progressively visible in the lateral ventricles as well. Our data strongly suggest that the hydrocephalus phenotype in *Pkd1* mutant mice originates from the third ventricle, the first compartment to develop ciliated ependyma, and subsequently expands to the lateral ventricles. In agreement with the phenotype observed in ubiquitously mutant embryos, we found that the third ventricle was massively enlarged at P8 in newborn mice carrying conditional inactivation of the *Pkd1* gene in brain. In this case, however, the lateral ventricles were also subsequently enlarged at P10, further suggesting a progressive dilatation of all the ventricles initiating from the third ventricle. Unfortunately, the Nestin-Cre model employed in this study is also known to be active in the kidney [33], [42]. The *Pkd1* ^Flox/−^;Nestin-Cre mice develop massive renal cystogenesis after birth, eventually causing death of the mice due to renal failure by day P12 (not shown), thus preventing us from studying the effect on hydrocephalus at later time points. However, we would predict that the phenotype becomes more severe with time. Future studies using a faithful brain-specific Cre that also targets the ependyma and choroid plexus will be necessary to clarify this aspect.

Interestingly, we found no major structural ciliary defects in the brains of these mice. This situation is different from all previously reported mice showing hydrocephalus due to a defective ciliary protein [33]–[37]. These data are in agreement with the previously proposed role of PC-1 in cilia, which was shown to be dispensable for a correct ciliogenesis, but necessary for ciliary function at least in the kidneys [27], [28]. In addition, our data showing no massive defects in the apico-basal polarity of the epithelium in the choroid plexus are also in agreement with the model previously proposed for the role of cilia in CSF formation [34], [37]. Previous studies on the role of Polaris (IFT88) in preventing hydrocephalus showed similar results. In that case, differences in the secretory properties of the choroid plexus cells were shown in addition to defective ependymal cilia motility, demonstrating that defective ciliary beating is not the sole explanation for hydrocephalus due to defective ciliary function [34], [37]. These data were further supported by the fact that hydrocephalus in the *Tg737* mouse develops prior to the initiation of ependymal cilia beating (postnatal life) [34]. Identical conclusions could be drawn from our results.

The function of cilia in most tissues is still a mystery. However, several studies have proposed a role for cilia in the regulation of different signalling pathways [38]. Therefore, in the ventricular system of the brain, it is conceivable that cilia might be important signalling platforms to allow precise control and coordinate the rate of secretion, movement, and re-absorption of the CSF. PC-1 is a large receptor able to regulate different signalling pathways. Thus, its function in the brain cilia might be to integrate this information in order to achieve the correct regulation of fluid balance.

## Materials and Methods

### Antibodies

The antibodies (Abs) used in this study were as follows: anti-c-Myc (Roche, 1667203, Cell signalling, 2272), anti-HA (Roche, 1867431), PC-2 (Santa Cruz Biotechnology, sc28331); anti-GAPDH (Cell Signalling Technologies, 2118) (1∶1000), anti-acetylated-α-tubulin (Sigma, T6793, 1∶1000). For western blot analysis horseradish peroxidase-conjugated secondary Abs (Amersham Bioscience, 1∶8000) were used and detected by ECL (GE Healthcare) or SuperSignal West Femto Maximum Sensitivity Substrate (Pierce Cat #34095). For immunofluorescence, secondary Abs conjugated with Alexa Fluor-488 (Molecular Probes, A21200, 1∶2000) and Alexa Fluor-594 (Molecular Probes, A21209, 1∶1000). All Abs were used in 3% BSA.

The anti-PC-1 antibodies used in this study were previously described [21]. They are both polyclonal antibodies generated in chicken (anti-CC) or rabbit (anti-LRR) by immunization using recombinant fragments of PC-1 corresponding to the entire C-tail or to the N-terminal LRR repeats, respectively [21].

### Ethical approval of all animal work

All animal care and experimental protocols, including the generation of chimeras, were conducted in accordance with the guidelines provided by the Italian Ministry of Health, upon approval of a specific protocol (IACUC-303) by the institutional care and use ethical committee (I.A.C.U.C.) at the San Raffaele Scientific Institute. Personnel from our own laboratory carried out all aspects of the mouse work under strict guidelines to insure careful, consistent and ethical handling of the mice.

### Generation of knock in/out mouse line

Transgenic ES cell lines were established using standard methods with the targeting vectors HALM and MLHA (Supporting Information) and injected in C57BL/6 blastocysts to generate chimeras. F1 knock-in *Pkd1^HAneo/+^* and *Pkd1^Mycneo/+^* mice (C57BL/6129Sv) were crossed with Flp-expressing mice [47] to excise the neomycin cassette. To get a pure genomic background, the knock-in lines were crossed with wild-type C57BL6 mice for seven further generations. Knock-out animals were generated by breeding knock-in lines with ubiquitous ATCB-Cre expressing mice [48] or by crossing knock in/out lines with Nestin-Cre mice [42].

### Genotyping and MEF cells isolation and culture

Genomic DNA was isolated from tails of mice and embryos as well as MEF cells. Knock-in mouse genotyping was performed by PCR using a three primer strategy (Supporting Information) to distinguish between wild-type, heterozygous, and homozygous knock-in and knock-out mice, respectively. PCR products were analysed on agarose gels. Mouse embryonic fibroblasts (MEF) were isolated from 11.5 day-old embryos as previously described [13], and cultured in DMEM, 10% FCS, 100 U/ml penicillin G sodium, and 100 µg/ml streptomycin sulphate.

### Histology and Immunofluorescence

Kidneys and brains from newborns and embryos were isolated, incubated in 20% sucrose/PBS overnight, and treated stepwise (6 to 14 hours, 4°C) with 30% sucrose, 10% glycerol/30% sucrose, and 15% sucrose/50% Killik solution (Bio Optica). After embedding in Killik and freezing in dry ice, samples were sectioned in the coronal plane.

For histological analysis, sections were stained with 10% Harris Hematoxylin (Sigma Aldrich) and 1% Eosin-G solution (Bio Optica). For immunofluorescence assays, sections were permeabilised (0.1% Triton X-100) and incubated in blocking solution (3% BSA/5% goat serum) and subsequently with primary Abs overnight at 4°C and secondary Abs for 1 hour at room temperature. After staining with DAPI (1∶10,000), sections were mounted in Mowiol/anti-fade solution (Invitrogen). Images were acquired and processed by Olympus IX70 DeltaVision RT Deconvolution System.

### Immunoprecipitation and immunoblotting

For immunoprecipitation studies tissues and cells were harvested and homogenised in lysis buffer (150 mM NaCl, 10% Glycerol, 1 mM glycerophosphate, 1 mM sodium orthovanadate, 1 mM sodium fluoride). After centrifugation, lysates were incubated for 1 hour at room temperature or overnight at 4°C with immobilised α-HA (agarose beads) or α-Myc Abs followed by incubation with G sepharose beads. After washing the beads with lysis buffer, precipitated proteins were resolved in sample buffer, boiled, separated by 4%, 3–8% or 4–12% gradient NuPAGE pre-cast gels (Invitrogen), transferred onto PVDF membranes, and analysed with the indicated primary and secondary Abs.

## Supporting Information

Figure S1 Generation of Pkd1 knock-in ES cells. (A) Genomic map and targeting strategy. The targeting vectors MLHA and HALM containing loxP sites, HA as well as myc tags and a neo cassette flanked by FRT sites, were transfected in ES cells for targeting the Pkd1 locus. After establishing of heterozygous knock in Pkd1Myc/+ and Pkd1HA/+ lines, the neo cassette was excised by crossing with Flpe expressing mouse. Single letters describe restriction sites in the Pkd1/Tsc2 genomic region: (E) EcoRI, (X) XbaI. (B) and (C) Southern blot analysis: Genomic DNA from (B) HALM and (C) MLHA targeted and selected ES cell clones were digested by XbaI and EcoRI, respectively. The XbaI digested DNA was analyzed with a probe against integrated tags in the 3′-end of the Pkd1 exon 46. A single band of 4.6 kb revealed the correct targeting of the ES cell clone. By analysis of EcoRI digested DNA with a probe against the neo gene, the correct integration was confirmed by the occurence of a single band of 11.6 kb.
(0.23 MB TIF)

Figure S2 Generation of homozygous knock-out mice. Heterozygous Pkd1+/ΔC mice were inter-crossed, and knock-out as well as wild-type embryos and dissections of embryos were analyzed. (B) Mutant mice develop edema and hemorrhage starting at E14.5, both of which are worsen at E15.5. (C) Tissue sections of embryos from E14.5 and E15.5 were stained with hematoxyline and eosine. Kidneys of homozygous Pkd1ΔC/ΔC embryos from E15.5 develop tubular as well as glomerular cysts. Barr represents 200 µm.
(0.79 MB TIF)

Figure S3 Specificity of the HA and Myc staining. Choroid plexus (CP) from mice Pkd1HA/HA (A) and control wt (B) at P2 were stained with anti-HA antibody (red) and with anti-acetylated tubulin antibody (green). Choroid plexus from mice Pkd1Myc/Myc (C) and control wt (D) at P2 were stained with anti-Myc antibody (red) and with anti-acetylated tubulin antibody (green). The arrows represent the signals for HA seen in the Pkd1HA/HA sections (A) and for myc Pkd1Myc/Myc sections (C) whereas no positive staining is observed in the wt sections (B and D). Barr represents 5 µm.
(0.60 MB TIF)

Table S1 Lethality of homozygous knock-out mice. For statistical purpose, heterozygous Pkd1+/ΔC mice were intercrossed, and embryos from E14.5 to E17.5 were genotyped by PCR and analyzed macroscopically. Heart beating was evaluated to establish dead versus alive embryos. A large proportion (59%) of homozygote Pkd1ΔC/ΔC mice survive up to E16.5, while their viability is completely absent at E17.5.
(0.02 MB DOC)

Methods S1 (0.03 MB DOC)
