# Supplementary figures and images for: A Novel Mouse Model Reveals that Polycystin-1 Deficiency in Ependyma and Choroid Plexus Results in Dysfunctional Cilia and Hydrocephalus
Source: PLoS One. 2009 Sep 23;4(9):e7137. doi: 10.1371/journal.pone.0007137 (PMC2743994; doi:10.1371/journal.pone.0007137)

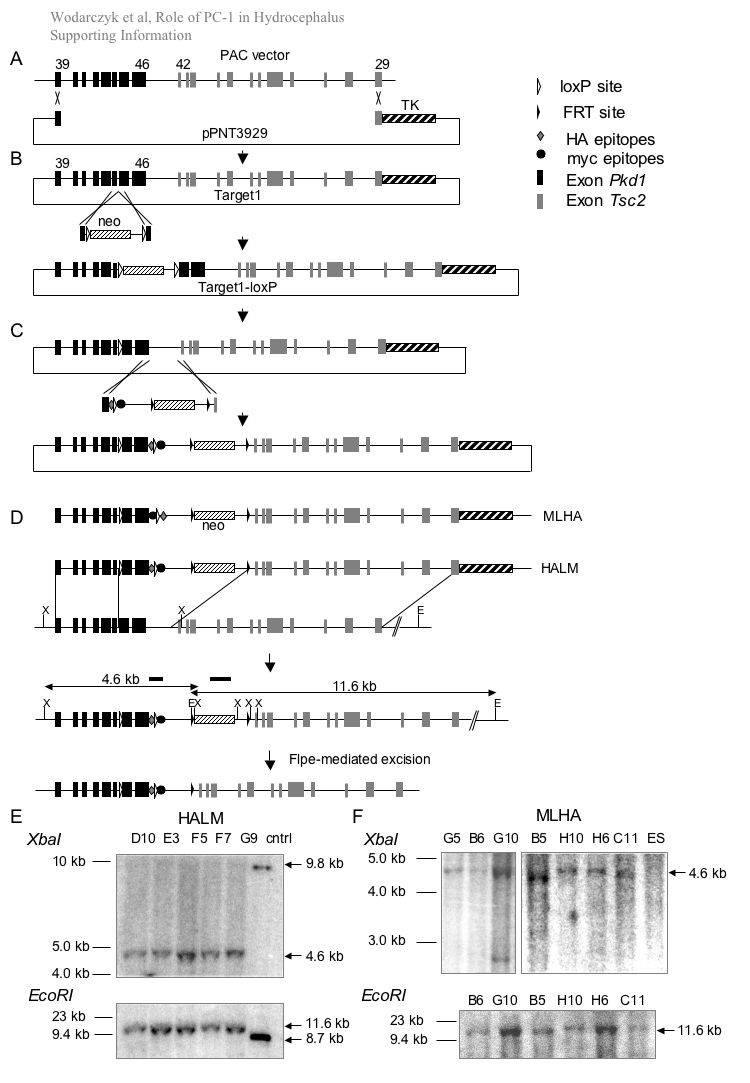

Supplement: Figure S1 — Generation of Pkd1 knock-in ES cells. (A) Genomic map and targeting strategy. The targeting vectors MLHA and HALM containing loxP sites, HA as well as myc tags and a neo cassette flanked by FRT sites, were transfected in ES cells for targeting the Pkd1 locus. After establishing of heterozygous knock in Pkd1Myc/+ and Pkd1HA/+ lines, the neo cassette was excised by crossing with Flpe expressing mouse. Single letters describe restriction sites in the Pkd1/Tsc2 genomic region: (E) EcoRI, (X) XbaI. (B) and (C) Southern blot analysis: Genomic DNA from (B) HALM and (C) MLHA targeted and selected ES cell clones were digested by XbaI and EcoRI, respectively. The XbaI digested DNA was analyzed with a probe against integrated tags in the 3′-end of the Pkd1 exon 46. A single band of 4.6 kb revealed the correct targeting of the ES cell clone. By analysis of EcoRI digested DNA with a probe against the neo gene, the correct integration was confirmed by the occurence of a single band of 11.6 kb. (0.23 MB TIF) [file pone.0007137.s001.tif]

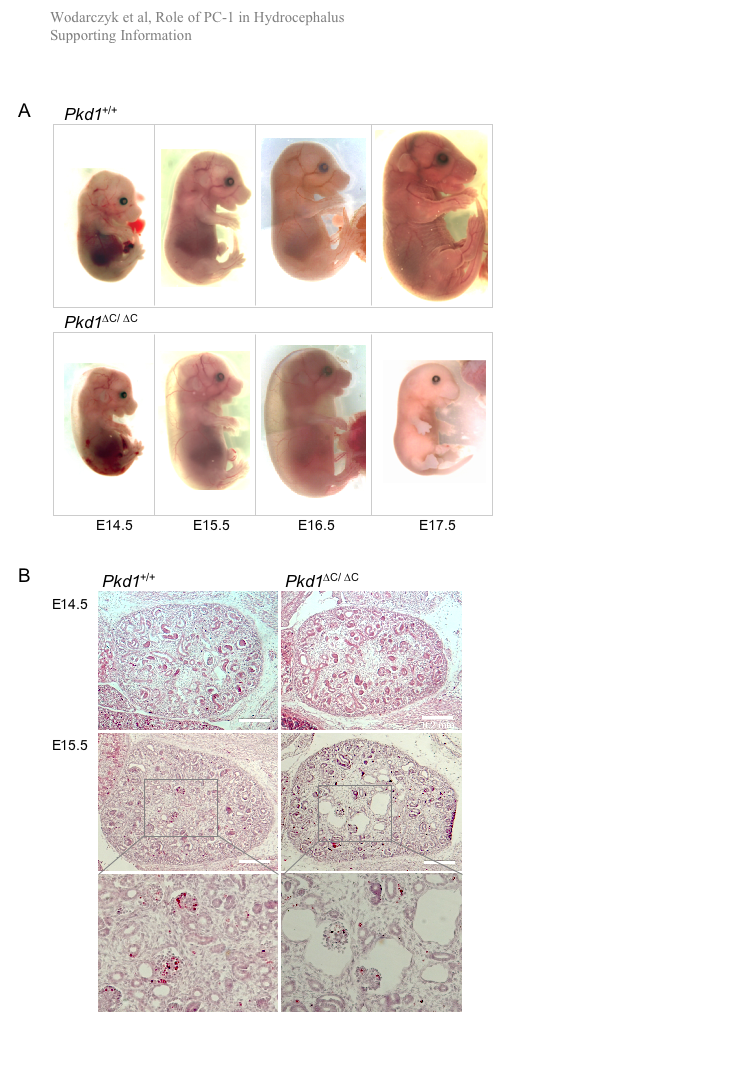

Supplement: Figure S2 — Generation of homozygous knock-out mice. Heterozygous Pkd1+/ΔC mice were inter-crossed, and knock-out as well as wild-type embryos and dissections of embryos were analyzed. (B) Mutant mice develop edema and hemorrhage starting at E14.5, both of which are worsen at E15.5. (C) Tissue sections of embryos from E14.5 and E15.5 were stained with hematoxyline and eosine. Kidneys of homozygous Pkd1ΔC/ΔC embryos from E15.5 develop tubular as well as glomerular cysts. Barr represents 200 µm. (0.79 MB TIF) [file pone.0007137.s002.tif]

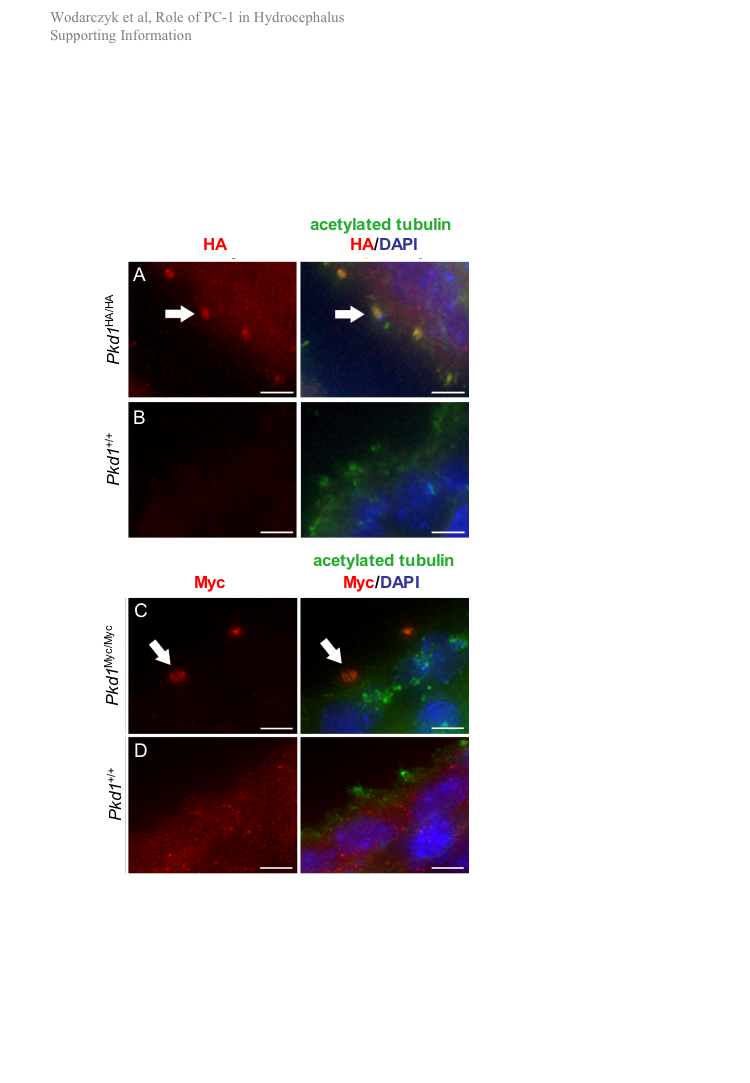

Supplement: Figure S3 — Specificity of the HA and Myc staining. Choroid plexus (CP) from mice Pkd1HA/HA (A) and control wt (B) at P2 were stained with anti-HA antibody (red) and with anti-acetylated tubulin antibody (green). Choroid plexus from mice Pkd1Myc/Myc (C) and control wt (D) at P2 were stained with anti-Myc antibody (red) and with anti-acetylated tubulin antibody (green). The arrows represent the signals for HA seen in the Pkd1HA/HA sections (A) and for myc Pkd1Myc/Myc sections (C) whereas no positive staining is observed in the wt sections (B and D). Barr represents 5 µm. (0.60 MB TIF) [file pone.0007137.s003.tif]
