# Supplementary material for: A Novel Mouse Model Reveals that Polycystin-1 Deficiency in Ependyma and Choroid Plexus Results in Dysfunctional Cilia and Hydrocephalus
Source: PLoS One. 2009 Sep 23;4(9):e7137. doi: 10.1371/journal.pone.0007137 (PMC2743994; doi:10.1371/journal.pone.0007137)
Supplement: Table S1 — Lethality of homozygous knock-out mice. For statistical purpose, heterozygous Pkd1+/ΔC mice were intercrossed, and embryos from E14.5 to E17.5 were genotyped by PCR and analyzed macroscopically. Heart beating was evaluated to establish dead versus alive embryos. A large proportion (59%) of homozygote Pkd1ΔC/ΔC mice survive up to E16.5, while their viability is completely absent at E17.5. (0.02 MB DOC) [file pone.0007137.s004.doc]

|  | ***Pkd1*^∆C/∆C^** | | ***Pkd1*^∆C/+^** | ***Pkd1*^+/+^** | **total** |
| --- | --- | --- | --- | --- | --- |
|  | alive | dead |  |  |  |
| E14.5 | 20 (100%) | 0 | 52 | 23 | 95 |
| E15.5 | 12 (92%) | 1 | 21 | 10 | 44 |
| E16.5 | 10 (59%) | 7 | 35 | 10 | 62 |
| E17.5 | 0 (0%) | 3 | 15 | 4 | 22 |
