# Supplementary material for: A Novel Mouse Model Reveals that Polycystin-1 Deficiency in Ependyma and Choroid Plexus Results in Dysfunctional Cilia and Hydrocephalus
Source: PLoS One. 2009 Sep 23;4(9):e7137. doi: 10.1371/journal.pone.0007137 (PMC2743994; doi:10.1371/journal.pone.0007137)
Supplement: Methods S1 — (0.03 MB DOC) [file pone.0007137.s005.doc]

## Supporting Methods

# Construction of the targeting vectors

The targeting vectors HALM and MLHA were stepwise built up by homologous recombination in bacteria (39). First, a PAC vector consistsing of the genomic sequence between *Pkd1* exon 39 and *Tsc2* exon 29, was identified by PCR screening of the mouse PAC library RPCI-21 using primers against *Pkd1* Exon 35/36 (fwd: GTC CAG TGT TCC TGG AGA, rev: AAG AAA GGA GGA CAG GAA), *Pkd1* exon 39/40 (fwd: CTA CGG CAG GTG CGA CTG, rev: GCA CCA CAC TCT GC), *Pkd1* exon 46 (fwd: GTC CGC TTT GAA GGA ATG, rev: GGC CTA CAG TCT GAT CCA), *Tsc2* exon 36/37 (fwd: TTG TGT TCC TAC AGC TCT, rev: TCT TGT GAG TGT CAT AGG) and *Tsc2* exon 29 (fwd: GGC CAT GGC CTT CGA, rev: CTG TGG GTC TGC GGA C). A PAC vector containing at least the genomic sequence between Pkd1 exon 39 and Tsc2 exon 29 was isolated and transformed in EL350 bacteria (39).

The recombination process was initiated resulting in an integration of the genomic region between *Pkd1* exon 39 and *Tsc2* exon 29 from the PAC vector into pPNT3929 vector (pre-targeting construct Target1). To integrate the first loxP site into the *Pkd1* intron 44, the mini targeting vector pl451 (39) was modified by flanking the internal kanamycin/neo hybrid cassette with sequences homologous to *Pkd1* intron 43/exon 44/intron 44 as well as *Pkd1* intron 44/exon 45. The arms were constructed by PCR using the primers a) *SalI*-CAG GAC CAT CCA CAC CAC and *EcoRI-*AGG CCC TTC CCT GGG ATA, and b) *BamHI-*AGG CCC TTC CCT GGG ATA and *NotI*-TCA GTC CGG CTG CAC CCT. The PCR products were integrated in pl451 by stepwise ligation through *SalI/EcorI* and *BamHI/NotI* leading to the vector pl451E4445. Furthermore, this vector was equipped with a additional loxP site by integration of a PCR fragment amplified from pl452 (39) with the primers *EcoRI-*ATA ACT TCG TAT AAT G and CCG ATC CCA TGG TTT AG. The PCR product was ligated into the *EcoRI/AgeI* opened pl451E4445 vector. This new mini targeting construct was linearized by *SalI/NotI* and transformed in EL350 bacteria containing the vector Target1 (Supplementary Figure 1B). By homologous recombination, two loxP sites and the kanamycin/neo cassette were integrated into *Pkd1* intron 44. By induction of Cre expression in those recombined bacteria, the interregion between the loxP sites in intron 44 was excised resulting in remaining of a single loxP site in this region (Target1-loxP).

For inserting the tags and the second loxP site into the 3’ region of *Pkd1* exon 46, other two mini-tageting vectors were constructed. In the first step, the *Tsc2* 3’UTR and exon 42 were amplified from the PAC vector by using the oligomere AAC TCC ACG GGC CAG ATG TG flanked by *BstBI* restriction site and the sequence for a FRT site, and the primer *NotI*-ATC CGT GAA GAG GTG CAC TA. The PCR product and the vector pl451 were digested by *BstBI /NotI* and ligated leading to the vector pl451-FRTTsc2. In the next step, the *Pkd1* 3’UTR was amplified by PCR with the primers *SalI-*GCC CTA GGG GTC TTG GCC and *EcoRI*-cag aga gca gca cag aca g and inserted into the *SalI/EcoRI* digested pl451-FRTTsc2 construct. To integrate the tags and the loxP site in this vector, intermediate vectors were constructed: At first, a sequence of the 3’ end of *Pkd1* exon 46 was amplified by the primers *XhoI/NotI*-TCG ACG CTG AAG C and *SpeI/AgeI*-AGT GCT GCT GGG GTG GAC and inserted into pKS vector by ligation through *XhoI* and *SpeI* resulting in the vector pKS-E46. An oligomere with the sequences for the loxP site in the centre and 5’ flanking *AgeI/BclI* restricition sites and stop codons as well as 3’flanking *XmaI/BglII/SalI/SpeI* restriction sites was designed. By ligation through *AgeI/SpeI*, the oligomere was inserted in the pKS-E46 resulting in the vector pKS-E46loxP. Next, a sequence containing three HA tags, and the sequence for 5 myc tags were excised by digestion of a PC-1-HA and a PC-1-Myc vector (20) with *AgeI/BclI*. The fragments were inserted in the 5’ region of the loxP site in the *AgeI/BclI* linearized pKS-E46loxP vector. As next, both constructs were digested by *XmaI* and *BglII*, and the *AgeI/BclI* digested fragments with the sequences for HA and myc tags (opposite to the first tags) were integrated in the 3’ part of the loxP site. The sequences for *Pkd1* exon 46, tags and the loxP site, were excised from the vectors by *XhoI/SalI* and ligated in the *XhoI/SalI* linearized vector pl451-FRTTsc2. The final constructs were linearized by *XhoI/HindIII* and transformed in EL350 bacteria containing the vector Target1-loxP. By homologous recombination, tags and the second loxP site were integrated in the 5’ end of *Pkd1* exon 46. The FRT flanked neo cassette was integrated in the interregion between the 3’ UTRs of *Pkd1* and *Tsc2* (Supplementary Figure 1C). The resulting targeting constructs HALM and MLHA (Supplementary Figure 1D) were used for homologous recombination in ES cells

**Generation of transgenic ES cell lines**

Targeting vectors HALM and MLHA were linearized by *NotI* digestion and electroporated in ES cells (TBV2). Cells were selected with G418 (200μg/ml)/Gancyclovir (2μM). To identify targeted ES cells, genomic DNA of selected clones were isolated, and the targeting of the *Pkd1* locus was checked initially by PCR using the external forward primer Int38 (CAG AAA TGT GCT GGG ATA CC) binding in *Pkd1* intron 38, and the internal reverse primer loxP1 (CGA AGT TAT GAA TTC AGG CC targets the first loxP site) resulting in a 2246 bp PCR product. ES cell clones tested positively by PCR, were finally analyzed by Southern blot (Supplementary Figure 1E, F) with two different probes: An internal 5’ probe against the tags was produced by PCR using the forward primer 46probe (TAA CAA GGT CCA CCC CAG C) binding exon 46 of *Pkd1*, the reverse primer TagMR (GGC GTC GAC TCA TTA AGA TC) recognizing specifically the 3’ end of the ultimate epitope sequence of targeting vectors, and as template the respective construct HALM or MLHA, generating a PCR product of 385 bp in length. A 3’ probe (798 bp) against the neo gene was created by PCR with the forward primer neo5 (ATG GGA TCG GCC ATT GAA C) and the reverse primer neo3 (GAA CTC GTC AAG AAG CG) and the vector HALM as template. Genomic DNA from ES cells was digested by *EcoRI* and analyzed with the P^32^ labeled anti tag probe in Southern blot. In a similar way, *XbaI* digested genomic DNA was rechecked with the neo probe.

**Removal of the Neomycin Cassette**

By ES cell technology, the first knock-in lines *Pkd1*^HAneo/+^ and *Pkd1*^mycneo/+^ were generated. The neo cassette was excised by crossing with Flp expressing mice (46) resulting in heterozygous *Pkd1*^HA/+^:Flp and *Pkd1*^Myc/+^:Flp lines. The Flp mediated excision of the neo cassette was proved by PCR using the forward primer NeoEx5 (TGT CTG TGC TGC TCT CTG) binds in the 3’UTR of *Pkd1*, the internal neo primer NeoInt5 (TGG GCT CTA TGG CTT CTG), and the reverse primer NeoEx3 (GAG TTT GTG TGA GGC CAG) targets the 3’ UTR of *Tsc2*. A PCR product of 215 bp indicated the remaining of the neo gene in the genome whereas a 174 bp PCR product gave prove for the excision of neo by Flp. Re-crossing of Pkd1^HA/+^:Flp and Pkd1^myc/+^:Flp lines with wild type C57Bl6 mice caused the loss of Flp gene. This was checked by PCR using the primers Flpfwd (CAC TGA TAT TGT AAG TAG TTT GC) and Flprev (CTA GTG CGA AGT AGT GAT CAG G) amplifying a 725 bp PCR product in case of transmission of the flp gene.

**Genotyping**

For the genotyping of the knock-in mice, following primers were used: the forward primer Tag5 (CAC AAT GGA CCT CCT TCC TC) binds in *Pkd1* exon 46, and the reverse primer TagMR (see above) targets specifically the knock in allele resulting in a 519 bp PCR product. To distinguish heterozygous and homozygous animals, the reverse primer Tag3 (TCT GAG AGG CCA GTG TGA AG) targets the 3’UTR of Pkd1, was additionally used in PCR allowing the identification of the wild type allele in heterozygous mice (PCR product of 225 bp in length).

Knock out embryos were detected by PCR with the forward primer 43MR (TGC TGC TGT TTG CCC TAT AC) binding in *Pkd1* exon 43, and the reverse primer Tag3 amplifying primarily a 862 bp (*Pkd1*^∆CMyc^) or a 748 bp (*Pkd1*^∆CHA^) PCR fragment. To distinguish between homozygous and heterozygous knock-out animals, the additional primer Tag5 was used allowing in interplay with primer Tag3 the detection of the wild type allele (579 bp in length).
